# mTFkb: a knowledgebase for fundamental annotation of mouse transcription factors

**DOI:** 10.1038/s41598-017-02404-w

**Published:** 2017-06-08

**Authors:** Kun Sun, Huating Wang, Hao Sun

**Affiliations:** 1 Li Ka Shing Institute of Health Sciences, The Chinese University of Hong Kong, Hong Kong SAR, China; 2 Department of Chemical Pathology, The Chinese University of Hong Kong, Hong Kong SAR, China; 3 Department of Orthopaedics and Traumatology, The Chinese University of Hong Kong, Hong Kong SAR, China

## Abstract

Transcription factors (TFs) are well-known important regulators in cell biology and tissue development. However, in mouse, one of the most widely-used model species, currently the vast majority of the known TFs have not been functionally studied due to the lack of sufficient annotations. To this end, we collected and analyzed the whole transcriptome sequencing data from more than 30 major mouse tissues and used the expression profiles to annotate the TFs. We found that the expression patterns of the TFs are highly correlated with the histology of the tissue types thus can be used to infer the potential functions of the TFs. Furthermore, we found that as many as 30% TFs display tissue-specific expression pattern, and these tissue-specific TFs are among the key TFs in their corresponding tissues. We also observed signals of divergent transcription associated with many TFs with unique expression pattern. Lastly, we have integrated all the data, our analysis results as well as various annotation resources to build a web-based database named mTFkb freely accessible at htt﻿p://www.m﻿yogenesisdb.org/mTFkb/. We believe that mTFkb could serve as a useful and valuable resource for TF studies in mouse.

## Introduction

Transcription factors (TFs) are a family of proteins that could bind to specific DNA sequences, usually in enhancer or promoter regions, to regulate the expression of target genes, either positively (as an activator) or negatively (as a repressor)^1–3^. In human, around 8% of the total genes encode TFs^4^. TFs are found to be highly conserved among most of the organisms. For instance, the numbers of annotated TFs in human (Homo Sapiens) and mouse (Mus Musculus) are similar^5^ and most of them are conserved between these two species. This highly conserved characteristic suggests that TFs are among the fundamental proteins for normal cellular functions^6^. Therefore, there is ongoing interest in the functional investigation of TFs. They are known essential regulators in normal cell function and tissue development. For instance, MyoD (Myogenic Differentiation 1) and Myf5 (Myogenic factor 5) play key roles in the development of limb and skeletal muscle^7, 8^. Furthermore, TFs that are key to guide cell differentiation and tissue development are discovered to interact with regulatory DNA elements such as enhancers and promoters^3, 9^. Recent studies also showed that key TFs could establish super-enhancers, clusters of enhancers with high activity, which are essential in controlling cell identity and disease^10, 11^. In addition, more and more studies demonstrated the successful reprogramming of somatic cells using a “cocktail” containing key TFs of the target cell type^12^. Very interestingly, emerging reports demonstrated the biological phenomenon of divergent transcription from the promoters of TFs^13, 14^, which could be helpful in deciphering its significance and functional mechanism^14, 15^. For instance, our group has recently discovered a novel long noncoding RNA, Linc-Yy1, which is transcribed from ~2 kb upstream of the Yy1 (Yin Yang 1) gene and serves as an important regulator of mouse skeletal myoblast differentiation through interaction with the Yy1 transcription factor^14^. Collectively, the existing studies reinforced that the TFs are among the most important regulators affecting the identity of cell/tissue type through diversified mechanisms of actions; it is thus imperative to identify the key TFs that are critical for the development of certain tissues.

Knowing their functional significance, however, most of the known TFs have yet to be characterized^16^. Existing studies in human found that the TFs are expressed in a tissue-dependent manner hence the expression pattern of the TFs across various tissues is closely correlated with their functions and could be used to mine the key TFs for the tissues^16–19^. Similar study however is still lacking in mouse, warranting the creation of a public knowledgebase for mouse TFs, which provides fundamental annotations. In addition, despite the existence of several TF databases such as TFdb^20^, TFCat^21^ and DBD^22^ that provide catalogs of TFs, functional characterizations are mostly lacking in these databases. RegNetwork^23^, YY1TargetDB^24^ and TFBSshape^25^ integrated only the regulatory targets information of the TFs. Another widely-used TF database, AnimalTFDB^5^, on the other hand, integrates annotations including Gene Ontology and regulatory pathway. In its 2.0 version^26^, it also incorporated tissue expression data but a limited number of mouse tissues were included with no further analyses provided. We reason that a database integrating expression analyses as well as functional annotations is needed to facilitate the studies on mouse TFs. To this end, in this study, we employed the transcriptome data from more than 30 major mouse tissues to annotate the TFs. Our analysis identified *bona fide* key TFs in many mouse tissues and shed novel insights of their tissue-specific functionality. In addition, divergent transcription associated with the promoter/enhancer regions of many TFs was observed and also showed unique tissue-specific expression pattern. Furthermore, we integrated functional annotations from various resources including protein-protein interactions, Gene Ontology (GO) and regulatory pathways to develop a web-based database named mTFkb (mouse transcription factor knowledgebase) freely accessible to the academic community, which we believe will become a valuable resource for studying TFs in mouse.

## Results

### The mouse transcription factor knowledgebase

**Figure 1:**
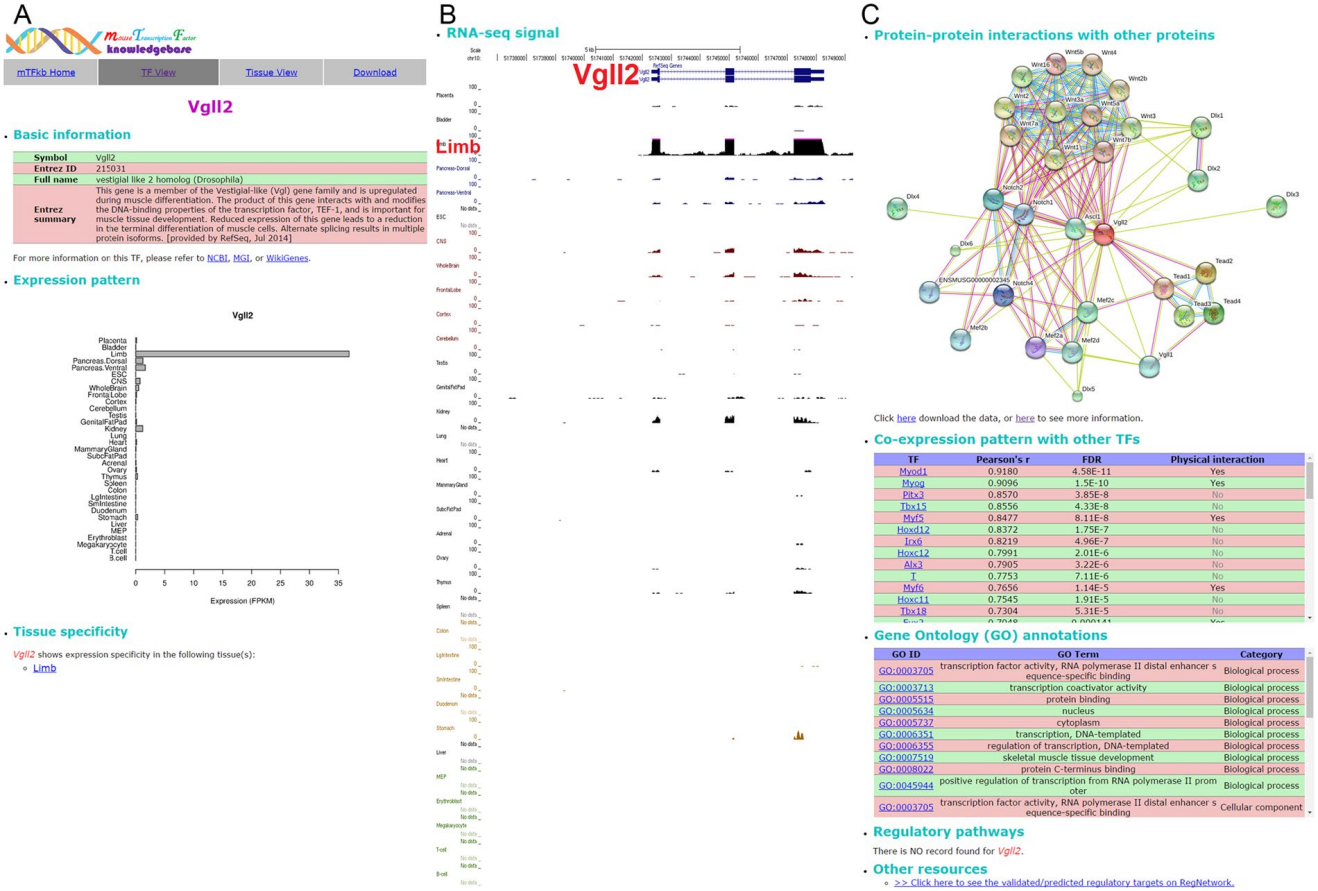
Snapshot of mTFkb webserver. Illustrated as an example is the transcription factor (TF) Vgll2. (**A**) Basic annotation, expression pattern and tissue-specificity identification; (**B**) Normalized RNA-seq signal across the mouse tissues analyzed; (**C**) Functional annotations of the TF including protein-protein interactions, co-expression pattern, Gene Ontology, regulatory pathways and targets.

We collected the whole transcriptome shotgun sequencing (a.k.a. RNA-seq) data for 33 major mouse tissues from the literature and profiled the expression pattern of 1,603 known mouse TFs (see Methods). Based on this data, we built a web-based database named mTFkb, which integrated all the expression data, our functional analysis results as well as functional annotations from various resources, freely available at http://sunlab.cpy.cuhk.edu.hk/mTFkb/. The database allows the users to inspect the expression profile and the analysis results for each mouse TF (the “TF View” page) or tissue (the “Tissue View” page) via a user-friendly interface. One snapshot was shown in Fig. 1 using Vgll2 (vestigial like 2 homolog (Drosophila)) as an example. Its basic information, expression pattern and tissue specificity can be fetched through the query function in the “TF View” page (Fig. 1A). Snapshot of RNA-seq signal tracks from each tissue is also included (Fig. 1B). Furthermore, we provide functional annotations including protein-protein interactions^27^, Gene Ontology (GO)^28^, and regulatory pathways^29^ as well as other possible information (e.g. regulatory targets^23–25^ if available) by integrating annotations from various resources (Fig. 1C). The detailed descriptions are provided in the following sections.

### Expression pattern of the mouse TFs

**Figure 2:**
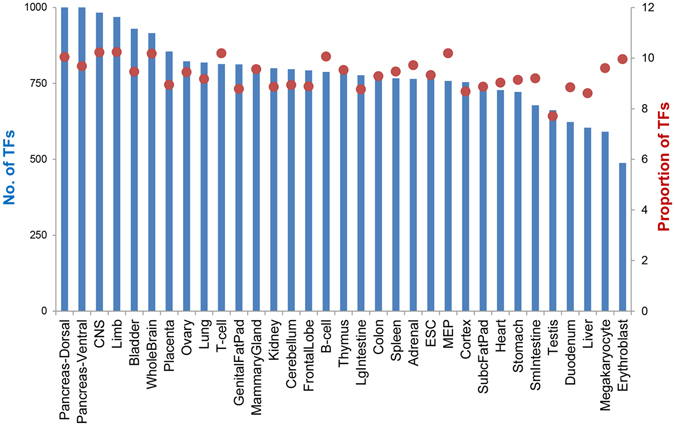
Numbers of transcription factors (TFs) expressed in each tissue (blue bars) and the proportion of the expressed TFs versus all expressed genes (red points, numbers are given as a percentage).

After profiling the expression values of the TFs using the RNA-seq data, we further investigated the expression pattern of the TFs across various tissues, which was also included in the “TF View” page. We found that the number of expressed TFs varies significantly among different tissues (Fig. 2, and the “Tissue View” page). For example, there were more than 1000 TFs expressed in pancreas tissues, while as a contrast, the number of expressed TFs in erythroblasts was only round 500. Still, when compared to the total number of genes expressed in each tissue, the proportions of the TFs were relatively stable (Fig. 2), which was consistent with previous findings in human^16^.

**Figure 3:**
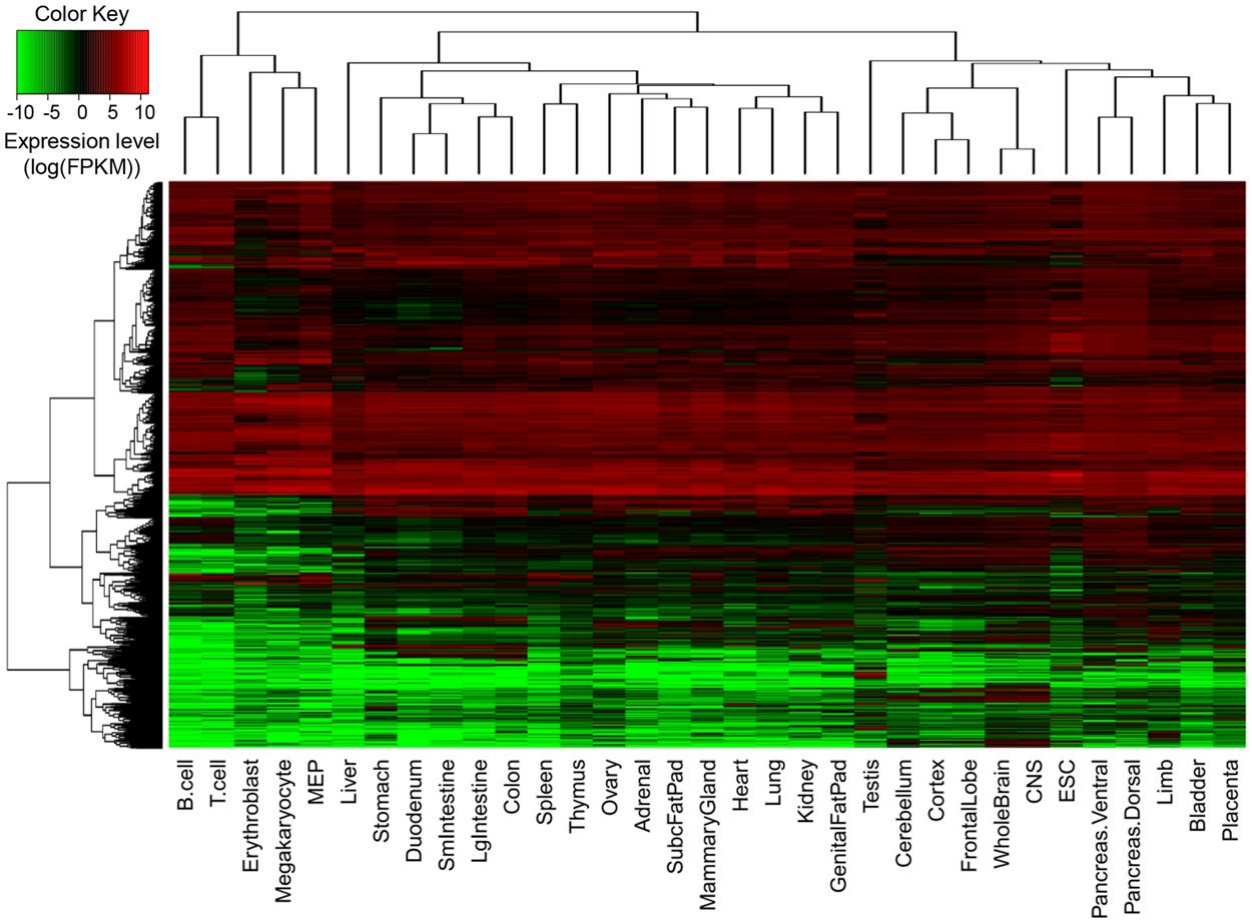
Hierarchical clustering of the mouse tissues using the expression values of the transcription factors.

**Figure 4:**
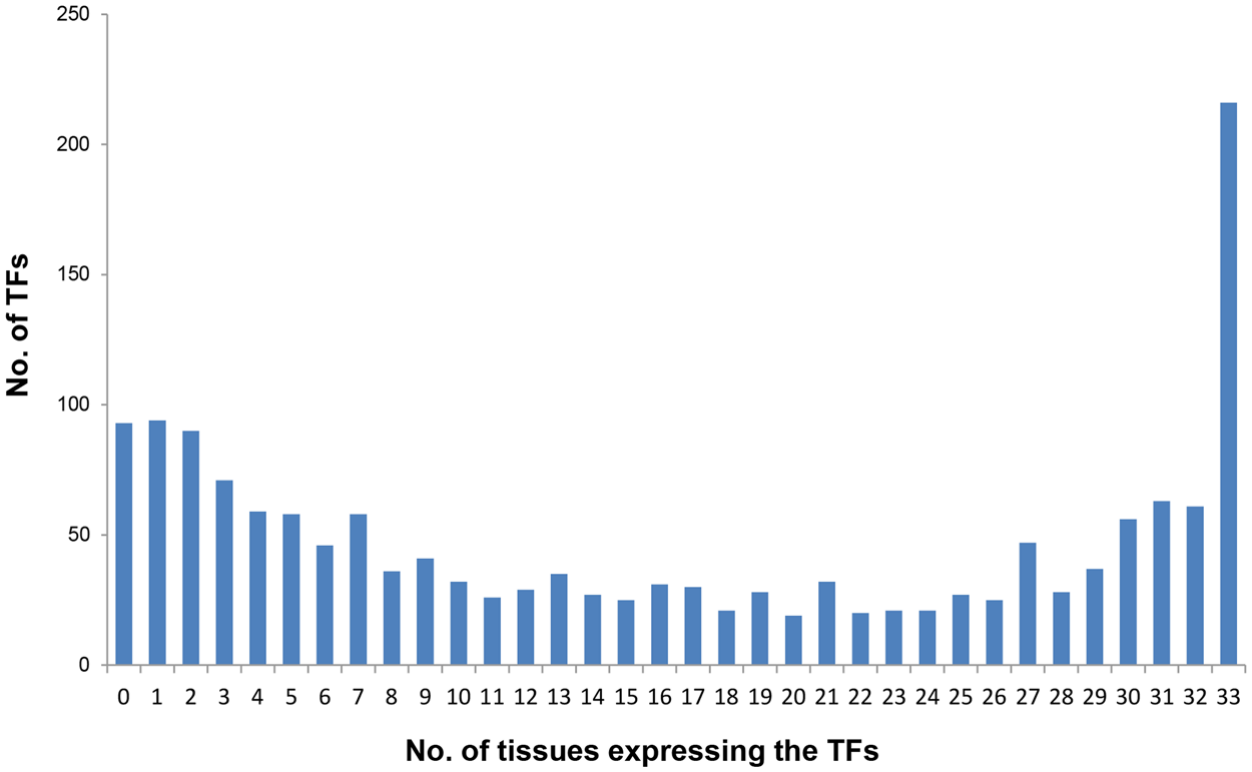
Distribution of transcription factors (TFs) based on the number of tissues in which they express.

Meanwhile, we performed the hierarchical clustering of the tissues using the expression values of the TFs. As expected, the result in Fig. 3 showed that histologically related tissues were clustered together. For instance, the tissues from hematopoietic system (B-cells, T-cells, Erythroblasts, Megakaryocytes and MEP (Megakaryocyte-Erythroid Progenitor cell)), digestive system (Stomach, Duodenum, Small intestines, Large intestines, and Colon), and nervous system (Cerebellum, Cortex, Frontal lobe, Whole brain, and CNS (Central Nervous System)) were clustered together, separately. This result indicated that the expression values of the TFs are highly correlated with the histology and function of the corresponding tissue. In addition, we also found that some TFs expressed ubiquitously in most tissues while others expressed in only a small proportion of the tissues. To strengthen the notion, for each TF, we counted the number of tissues in which it is expressed. As shown in Fig. 4, we found that TFs expressed in a “U-shape” across the tissues, i.e., the majority of the TFs tend to express in either most of the tissues or in a very limited number of tissues, suggesting diversified functional scenarios: some TFs are “housekeeping” while others may be highly tissue-specific. The latter are more likely to be key TFs defining and maintaining the cell/tissue identity^3, 10, 17^, thus deserved a more intensive exploration.

### Exploration of key TFs in various mouse tissues

**Figure 5:**
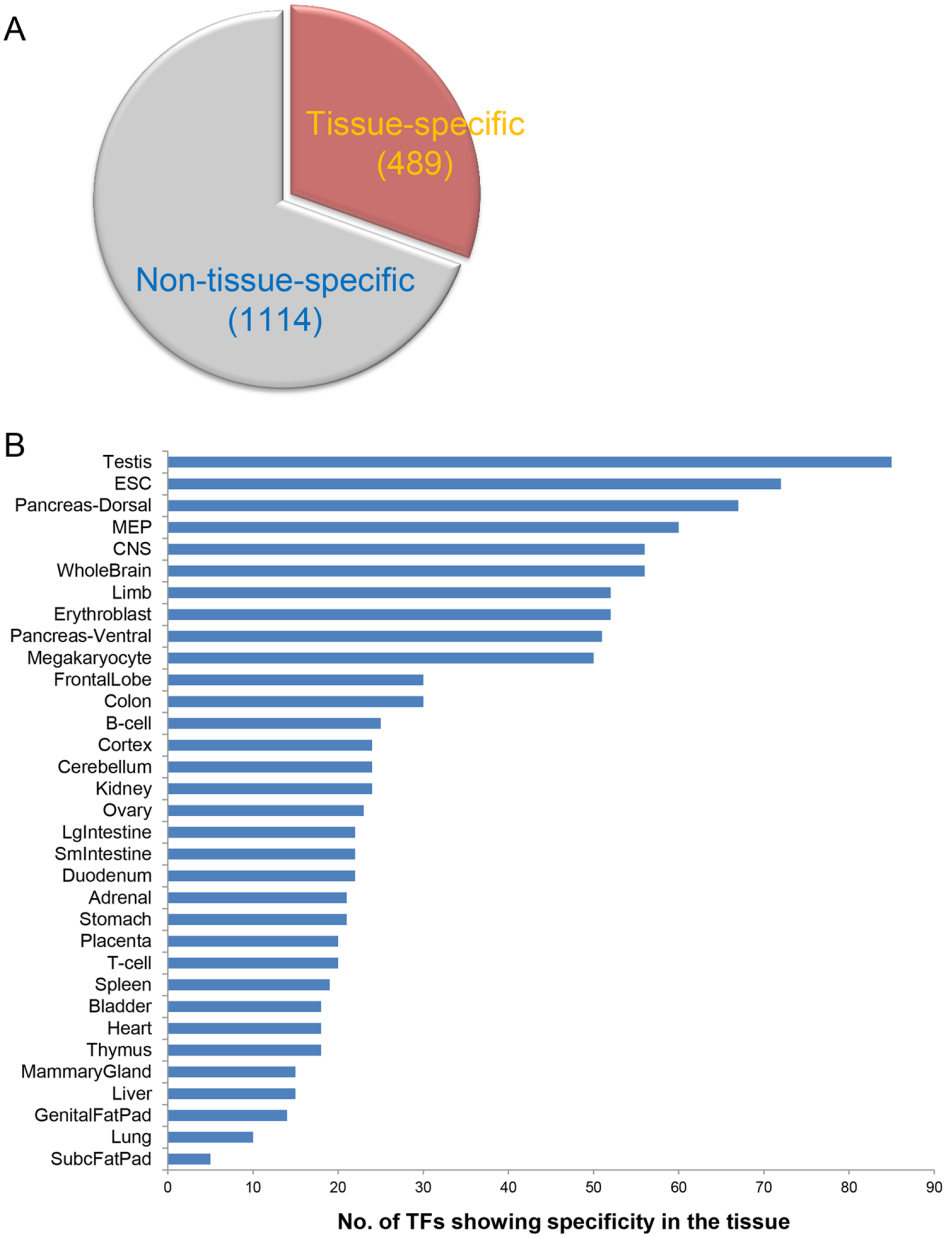
(**A**) 489 out of 1603 (30.5%) transcription factors (TFs) show tissue specific expression pattern while 1114 are non-tissue specific. (**B**) Number of tissue specific TFs in each mouse tissue.

As shown in Fig. 2, for all the tissue types, hundreds of TFs are expressed, while usually only a small proportion of them are potential key TFs which play important roles that are tightly related to the function and identity of the tissue type. As shown in Fig. 3 by the hierarchical clustering, we found that the dynamics of the TF expression are highly correlated with the tissue histology, the expression patterns was thus used to identify key TFs for the tissues^17^. To this end, we searched for tissue-specifically expressed TFs as the candidates key TFs (see Methods) (The “Tissue View” page). As a result, we found that around 30% (489 out of 1603) TFs showed tissue-specificity (Fig. 5A). On the other hand, the number of TFs that show specificity in each tissue type varies significantly (Fig. 5B). For instance, more than 70 TFs are specifically expressed in ESC while less than 20 in liver (Fig. 5B). The variation in the number of specifically-expressed TFs across the tissues might be correlated with the functional complexity of the tissues.

**Figure 6:**
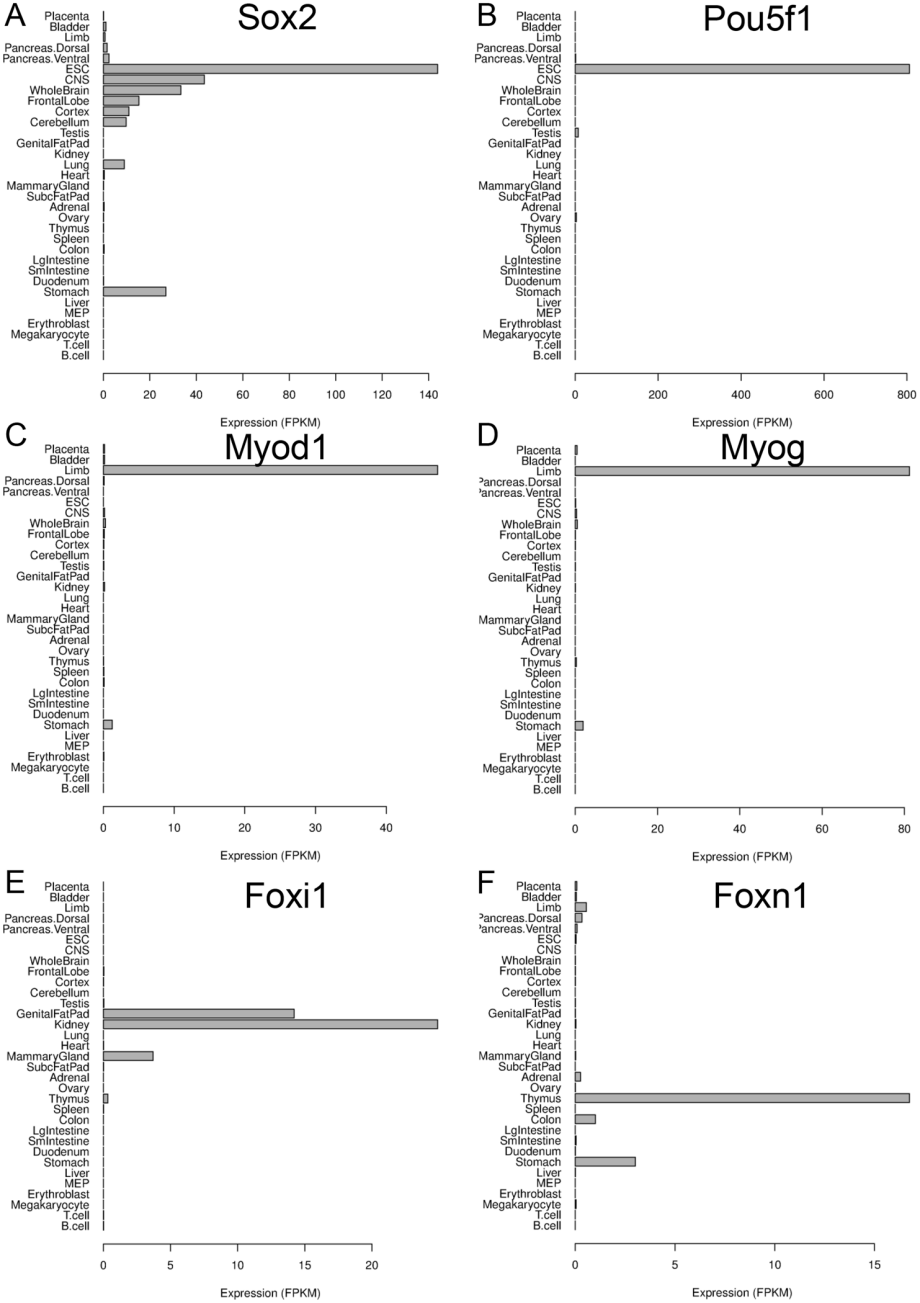
Expression pattern of selected known key transcription factors (TFs). (**A**) Sox2. (**B**) Pou5f1. (**C**) Myod1. (**D**) Myog. (**E**) Foxi1. (**F**) Foxn1.

To investigate the performance of our approach, especially the ability to identify potential key TFs, we first examined several well-known master TFs for certain tissues. As shown in Fig. 6, Sox2 (SRY-box containing gene 2) and Pou5f1 are known master TFs in ESC^30, 31^, and indeed we found them specifically expressed in ESC (Fig. 6A and B); similarly, Myod1 and Myog (myogenin) are known master TFs in skeletal muscle development^32^ and was found to display specificity in limb tissue (Fig. 6C and D); Foxi1 (forkhead box I1) and Foxn1 (foxhead box N1) were identified to be expressed in kidney and thymus, respectively, which is consistent with previous knowledge that they are key regulators of kidney^33^ and thymus^34^, respectively (Fig. 6E and F). In addition, we compared our result with the key TFs identified in human by D’Alessio *et al*.^17^. Interestingly, we found that in many tissues, the mouse orthologs of top-ranked key TFs in human were also identified to be specific in the homologous tissue in mouse. A comparison for the pancreas tissue (“pancreatic islet cells” in D’Alessio *et al*. versus “pancreas ventral” in mTFkb) was shown in Table 1 as an illustrating example and more results from other tissues could be found in Suppl. Table S1.

**Table 1:** Co-occurrence of key transcription factors in human and mouse pancreas tissues.

Rank in human pancreas	Transcription factor	Occurrence in mTFkb
1	RFX6	Yes
2	INSM1	No
3	PAX6	Yes
4	ISL1	Yes
5	NEUROD1	Yes
6	GLIS3	No orthologue in mouse
7	NR5A2	Yes
8	ZNF165	No orthologue in mouse
9	ARX	Yes
10	MNX1	Yes
14	MAFB	No
371	PDX1	Yes
543	PAX4	No
755	NEUROG3	Yes

**Figure 7:**
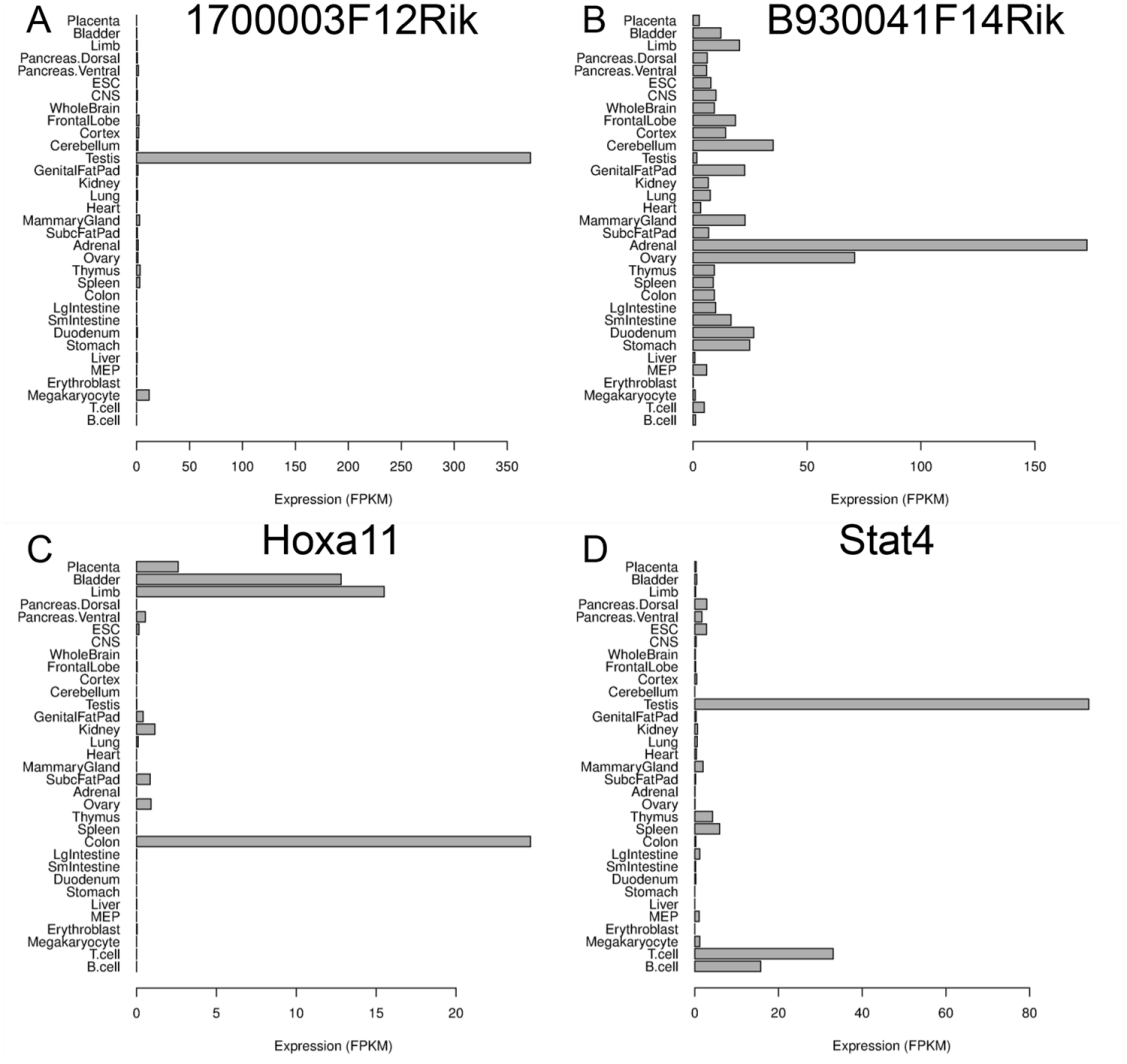
Expression pattern of selected transcription factors (TFs) with uncharacterized functions. (**A**) B930041F14Rik. (**B**) 1700003F12Rik. (**C**) Hoxa11. (**D**) Stat4.

The tissue-specificity identified by mTFkb might suggest uncharacterized functions of the TFs in their corresponding tissues and this information could be especially valuable for the TFs that have not been comprehensively investigated. For instance, 1700003F12Rik and B930041F14Rik are two TFs coded by RIKEN cDNA 1700003F12 and B930041F14 genes, respectively, and their functions remain completely uncharacterized. Our data revealed their unique expression in testis (Fig. 7A) and adrenal glands (Fig. 7B), respectively, which will be helpful in guiding the functional studies in the future. On the other hand, Hoxa11 (homeo box A11) is known to be involved in repressing MyoD during limb muscle development^35^. Interestingly, in addition to the high expression in limb, we found that it is also enriched in bladder and colon, which suggested potentially uncharacterized functions (Fig. 7C). To this end, the antisense gene of human HOXA11 (i.e., HOXA11-AS) was demonstrated to be a biomarker for urothelial carcinoma^36^ which also correlates with tumor size and metastasis in colorectal cancer^37^, supporting that Hoxa11 may play some roles in the bladder and colon tissues. Similarly, Fig. 7D shows that Stat4 (signal transducer and activator of transcription 4) is specifically expressed in the lymphocytes and testis. It is known to be essential for mediating responses to IL12 in lymphocytes and regulating the differentiation of T helper cells^38^, while its potential functions in the testis remain to be investigated. Our expression analysis thus provided valuable information for future functional and mechanistic studies.

### Divergent transcription associated with TFs

**Figure 8:**
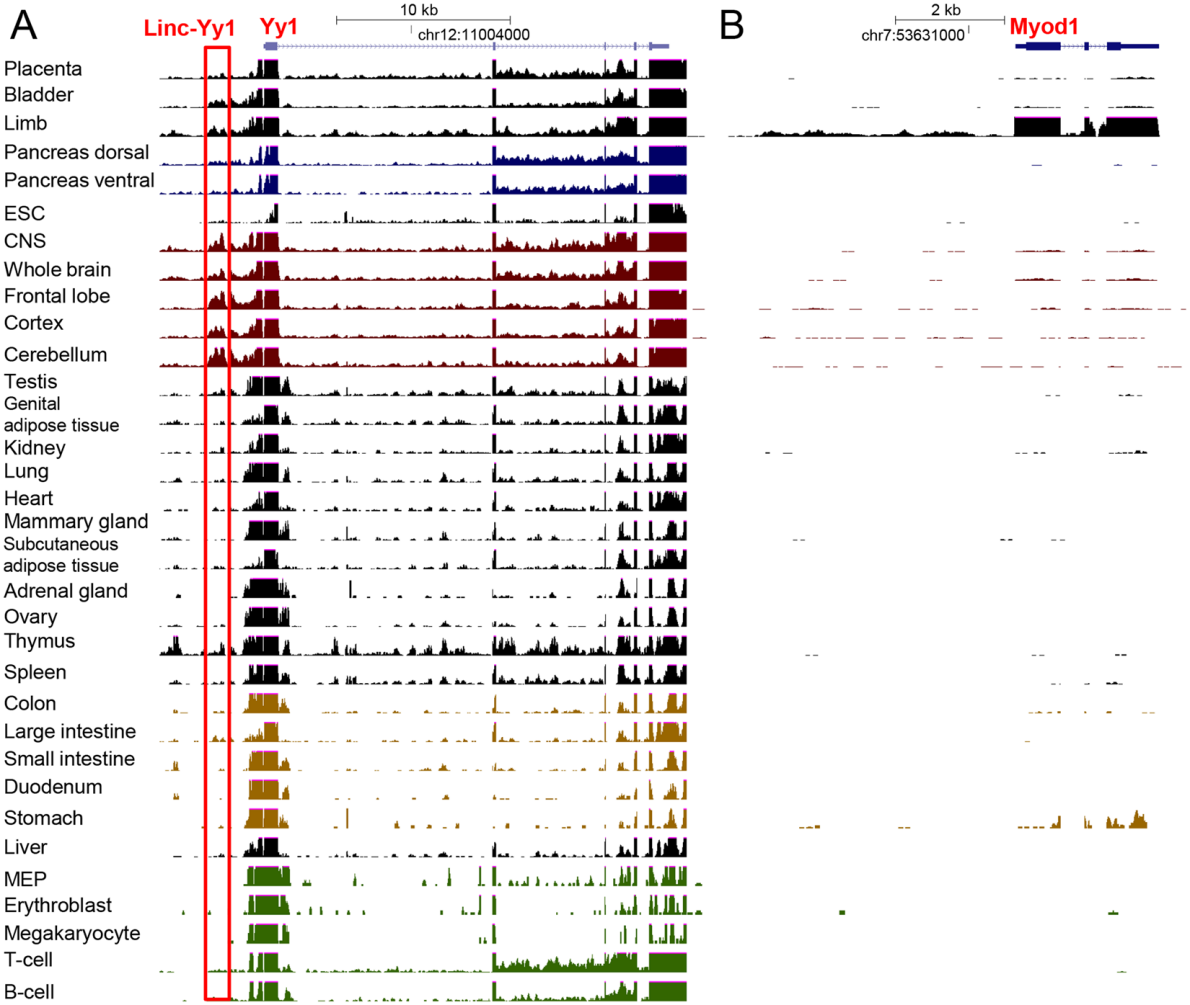
Normalized RNA-seq signal across the mouse tissues for (**A**) Yy1 and (**B**) Myod1. The red box in (**A**) indicates the genomic coordination of Linc-Yy1, the characterized divergent transcript of Yy1.

To explore whether divergent transcription associated with TFs is a prevalent phenomenon in mouse tissues, we further examined the normalized RNA-seq signals that could be obtained by querying the TF through the “TF View” page (Fig. 1B). For many TFs, we could observe a certain level of RNA-seq signal at the promoter/enhancer regions, indicating the potential existence of divergent transcription associated with the TFs. Yy1 and Myod1 were plotted in Fig. 8 as examples. Consistent with our recent report, there is strong RNA-seq signal for the divergent transcript of Yy1 gene (i.e., Linc-Yy1)^14^. However, despite the fact that Yy1 is ubiquitously expressed among most mouse tissues^24, 39^, the signal of Linc-Yy1 could only be observed in limb and the nervous system (Fig. 8A). The function of Yy1 and its interplay with Linc-Yy1 has been characterized in the muscle development^40^; it however remains to be determined whether Linc-Yy1 interacts with Yy1 during the development of nervous system considering that Yy1 is a known important regulator during the nervous system development^41^. Similarly, RNA-seq signal was also observed in the promoter of Myod1 in limb tissue where MyoD is highly expressed (Fig. 8B), which warrants further investigation in the future. Collectively, these findings suggested that divergent transcripts display unique tissue-specific expression pattern independent of the associated TFs.

### Functional annotations of the TFs

The above expression analysis, key TF annotation and divergent transcription have, to some degree, provided information on the functional aspects of each TF. To further strengthen the functional annotations, we analyzed various features of a TF, including protein-protein interactions^27^, co-expression pattern, Gene Ontology (GO)^28^, regulatory pathways^29^ as well as other annotation resources (e.g. regulatory targets^23, 24^ and DNA binding pattern^25^) by integrating existing information into mTFkb (Fig. 1C). As shown in Fig. 1A and B, analysis of RNA-seq data showed that Vgll2 is specifically expressed in the limb tissue, suggesting that it may serve as a key TF in the muscle system which is consistent with the previous knowledge of its involvement in skeletal muscle differentiation^42, 43^. To gain further understanding of its role, inspection of protein-protein interaction led to the discovery that Vgll2 interacts with a cluster of Wnt proteins, Mef2 (myocyte enhancer factor-2) family proteins (Mef2a, Mef2b, Mef2c and Mef2d), Notch1 and Notch2 as well as Tead (TEA domain) family proteins (Fig. 1C). Consistently, previous study had shown the interaction between Vgll2 and Mef2d in C2C12 cell line (a widely used mouse myoblast cell line)^44^; the interaction with the Wnt signaling pathway had also been discovered in Xenopus^45^. In addition, by co-expression analysis, we found that Vgll2 was associated with Myod1, Myog, Pitx3 (Paired Like Homeodomain 3), Tbx15 (T-box 15), Myf5, etc (Fig. 1C); among them physical interactions were also identified with Myod1, Myog and Myf5, which are well-known regulatory TFs of skeletal muscle development^32^, suggesting its possible functional connection with mouse skeletal muscle development. Consistently, GO analysis revealed a GO term of “skeletal muscle tissue development” related with Vgll2 (Fig. 1C). Exploring RegNetwork^23^, a data repository of regulatory relationships for human and mouse through “other resources”, we found no known regulatory targets of Vgll2 probably due to the lack of sufficient functional studies of Vgll2 in mouse (Fig. 1C). Our functional annotations thus had revealed unknown aspects of Vgll2 involvement in limb development that could be tested experimentally in the future.

## Discussion

In this paper, we present mTFkb, a web-based database dedicated to the annotation of mouse TFs. mTFkb integrates the expression data from 33 major mouse tissues and provides novel insights into the expression pattern of the TFs. mTFkb is freely available thus allowing users to inspect the data for any TF and tissue via the web interface. Unlike most of other TF databases that only provide the catalog of the TFs or limited functional information, mTFkb provides the fundamental functional annotations including the tissue-specificity identification, key TF interference, RNA-seq signal profiling, divergent transcription screening, protein-protein interaction, co-expression analysis, GO annotation as well as regulatory pathway/targets. In addition, mTFkb covers the major tissues in mouse therefore serves as a comprehensive and valuable resource for fundamental functional annotation of TFs.

Among all the functional analyses that mTFkb provides, the identification of key TFs is the most valuable. Inferring the functional importance of known and unknown TFs in certain tissues will be valuable to guide the selection of the most important TFs in the tissue of interest for future mechanistic investigations. These key TFs likely represent the functional components in the “cocktail” of TFs used for cell reprogramming^17^. Not surprisingly, we found that the key TFs identified in mTFkb highly resembled those predicted in human since most TFs are highly conserved between human and mouse. For example, we found that most of the co-occurred TFs in Table 1 had been proved to play key roles in the Pancreas tissue. For instance, Rfx6 (regulatory factor X6) and Pax6 (paired box 6) are essential to maintain the functional identity of pancreatic beta-cells^46^ and islet cells^47^, respectively; Isl1 (ISL LIM homeobox 1) is a well-known key regulator for pancreatic islets and functions in the maturation, proliferation and survival of the endocrine pancreas^48^. Collectively our data suggest that the tissue-specific TFs included in mTFkb are of high confidence to be the *bona fide* key TFs of the corresponding tissues.

Besides the expression pattern analysis, we also profiled divergent transcription associated with TFs. Unfortunately, due to the lacking of a comprehensive catalog of the divergent transcripts, a quantitative analysis could not be performed. Nevertheless, our findings suggested the wide existence of divergent transcription and distinct tissue-specific expression pattern from the associated TFs. For instance, MyoD is a tissue-specific TF for limb and the divergent transcription signal was only observed in limb (Fig. 8B). In contrast, even though Yy1 did not show strong tissue-specificity, its divergent transcript (i.e., Linc-Yy1) showed tissue-specific expression pattern (Fig. 8A). In this regard, the RNA-seq signal mTFkb provides could serve as a resource for inspecting the presence and expression pattern of the potential divergent transcription for further studies of their functionality.

Lastly, by integrating functional annotations from various existing resources, further analysis of a TF’s role through protein-protein interaction, co-expressed TFs, GO analysis and regulatory pathways/targets become possible. As demonstrated by the example of Vgll2 in Fig. 1, the integrated information can serve as the foundation for future functional exploration of a TF in certain tissue/cell.

## Materials and Methods

### Data collection

33 RNA-seq datasets, one for each mouse tissue, were collected from multiple sources including the ENCODE Project (Adrenal glands, Bladder, Cerebellum, CNS, Colon, Cortex, Duodenum, Frontal lobe, Genital adipose tissue, Heart, Kidney, Large intestine, Limb, Liver, Lung, Mammary Gland, Ovary, Placenta, Subcutaneous adipose tissue, Small intestine, Spleen, Stomach, Testis, Thymus, Whole brain, Erythroblast, Megakaryocyte and MEP)^49^, Guttman *et al*. (ESC)^50^, Kim *et al*. (T-cell and B-cell)^51^ and Rodriguez-Seguel *et al*. (Pancreas ventral and Pancreas dorsal)^52^. The detailed information could be found in Suppl. Table S2. For consistency, all the RNA-seq datasets involved in this study were generated using Poly-A extraction protocol and sequenced on Illumina sequencer.

A list of 1,675 mouse TFs was obtained from the most updated version of TFdb (Riken TF Database)^20^, which is a widely used database in the literatures. Among the 1,675 annotated TFs, 1,603 (95.5%) could be found in RefSeq^53^ gene annotation and used in the analyses.

### RNA-seq data processing and expression profiling

After downloading the raw sequencing reads from the RNA-seq datasets, a preprocessing procedure was first performed to remove 1) sequencing adaptors; 2) low-quality base-pairs; and 3) PCR duplications using in-house programs^54^. Then the filtered reads were aligned to the mouse reference genome (UCSC mm9/NCBI 37) using TopHat (version 2.0.9)^55^ guided by the RefSeq^53^ genes (the “-G” option of Tophat) with default parameters. Gene expression profiling was performed using Cufflinks (version 2.1.1)^56^ against the RefSeq genes with default parameters. Cufflinks employs a built-in normalization scheme to improve the estimation of expression^57^. The expression level of the genes were quantified as FPKM (Fragments Per Kilobase of transcript per Million mapped reads)^56^ values which had been demonstrated to be a reasonable measurement for expression quantifications^58^. A FPKM value of 5 was used as the threshold to call a gene/TF as “expressed” in each tissue type. A value of 1 was added to each raw FPKM value of the expression matrix before transforming to log2 scale (i.e., log-normalization) for the downstream data analyses^18, 19^. The above log-normalization method has been demonstrated to be an appropriate normalization method for tissue-specificity analysis^18^. Indeed, after the log-normalization, we found that the expression distributions were similar across the samples and the hierarchical clustering result was based on the tissue histology rather than the laboratory of origin, confirming that the normalized expression profiles appeared consistent and comparable across the samples^18, 57–59^. Hierarchical clustering of the tissues using expression values of the TFs was performed using R. For each tissue, the RNA-seq signal was extracted from the TopHat mapping result and normalized by the total number of aligned reads using in-house programs. For co-expression analysis, we calculated the Pearson’s correlation for all the TF pairs using the expression values across all the tissues and the p-values were further adjusted using the Bonferroni correction method.

### Identification of key TFs

We defined key TFs as those expressing in a tissue-specific manner and at a relatively high level in the corresponding tissues^17^. To identify tissue specifically expressed TFs, an algorithm adapted from Kadota *et al*.^60^ was employed. This algorithm considers the task of tissue-specific gene identification as an “outliner identification” problem. The main advantage of this algorithm is that objective decisions could be made because the procedure is independent of a significance level^60^. Basically, for each TF, its expression values among various tissues were collected; the tissue specific expression in certain tissue was identified as “outliners” compared to the remaining tissues. Next, considering that the key TFs should express at a relatively high level in the corresponding tissue, we further filtered out the candidate TF-tissue pairs in which the expression of the TF is low (a FPKM value of 10 was used as the cutoff) in the candidate tissue. The implementation of this algorithm is freely accessible on our website.

## supplementary material

Supplementary information

datasetS1

datasetS2
