# Supplementary material for: mTFkb: a knowledgebase for fundamental annotation of mouse transcription factors
Source: Sci Rep. 2017 Jun 8;7:3022. doi: 10.1038/s41598-017-02404-w (PMC5465081; doi:10.1038/s41598-017-02404-w)
Supplement: Supplementary file 1 — Supplementary information [file 41598_2017_2404_MOESM1_ESM.doc]

mTFkb: a knowledgebase for fundamental annotation of mouse transcription factors

Kun Sun, Huating Wang, and Hao Sun

**Supplementary information**

Table S1: Co-occurrence of key transcription factors in human and mouse tissues.

Table S2: Basic information of the RNA-seq datasets used in this study.
